# Etiologic reclassification of cryptogenic stroke after implantable cardiac monitoring and computed tomography angiography re-assessment

**DOI:** 10.1007/s00415-022-11370-x

**Published:** 2022-09-13

**Authors:** Francesco Mele, Giuseppe Scopelliti, Arianna Manini, Carola Ferrari Aggradi, Matteo Baiardo, Marco Schiavone, Maurizio Viecca, Andrea Ianniello, Pierluigi Bertora, Giovanni B. Forleo, Leonardo Pantoni

**Affiliations:** 1 Neurology Unit, Luigi Sacco University Hospital, Milan, Italy; 2 Department of Biomedical and Clinical Sciences, University of Milan, Via Giovanni Battista Grassi, 74, 20157 Milan, Italy; 3 Univ. Lille, Inserm, CHU Lille, U1172, LilNCog, Lille Neuroscience & Cognition, Lille, France; 4 Department of Pathophysiology and Transplantation, “Dino Ferrari” Center, University of Milan, Milan, Italy; 5 Cardiology Unit, Luigi Sacco University Hospital, Milan, Italy; 6 Radiology Unit, Luigi Sacco University Hospital, Milan, Italy

**Keywords:** Cryptogenic stroke, Esus, Atrial fibrillation, Nonstenotic carotid plaques, Aortic arch atherosclerosis

## Abstract

**Introduction:**

Different mechanisms may underlie cryptogenic stroke, including subclinical atrial fibrillation (AF), nonstenotic carotid plaques (NCP), and aortic arch atherosclerosis (AAA). In a cohort of cryptogenic stroke patients, we aimed to: (1) evaluate the prevalence of subclinical AF, NCP, and AAA, and reclassify the etiology accordingly; (2) compare the clinical features of patients with reclassified etiology with those with confirmed cryptogenic stroke.

**Methods:**

Data of patients hospitalized for cryptogenic stroke between January 2018 and February 2021 were retrospectively analyzed. Patients were included if they received implantable cardiac monitoring (ICM) to detect subclinical AF. Baseline computed tomography angiography (CTA) was re-evaluated to assess NCP and AAA. Since aortic plaques with ulceration/intraluminal thrombus were considered pathogenetic during the initial workup, only patients with milder AAA were included. Stroke etiology was reclassified as “cardioembolic”, “atherosclerotic”, or “mixed” based on the detection of AF and NCP/AAA. Patients with “true cryptogenic” stroke (no AF, ipsilateral NCP, or AAA detected) were compared with those with reclassified etiology.

**Results:**

Among 63 patients included, 21 (33%) were diagnosed with AF (median follow-up time of 15 months), 12 (19%) had ipsilateral NCP, and 6 (10%) had AAA. Stroke etiology was reclassified in 30 patients (48%): cardioembolic in 14 (22%), atherosclerotic in 9 (14%), and mixed in 7 (11%). Patients with true cryptogenic stroke were younger compared to those with reclassified etiology (*p* = 0.001).

**Discussion:**

One or more potential covert stroke sources can be recognized in half of the patients with a cryptogenic stroke through long-term cardiac monitoring and focused CTA re-assessment.

**Supplementary Information:**

The online version contains supplementary material available at 10.1007/s00415-022-11370-x.

## Introduction

Between 20 and 30% of all ischemic strokes have no definitively detected etiology at discharge, even after extensive diagnostic workup, and are thus labeled cryptogenic [1]. Recent studies have hypothesized different mechanisms possibly underlying cryptogenic stroke, including subclinical atrial fibrillation (AF), aortic arch atherosclerosis (AAA), and nonstenotic (i.e., causing a reduction of arterial lumen < 50%) carotid plaques (NCP) [2].

AF is a well-known cause of ischemic stroke, but its possibly erratic occurrence poses some difficulties in its detection, since standard in-hospital cardiac monitoring may not be sufficient to disclose brief arrhythmic episodes. Prolonged monitoring by means of an implantable cardiac monitor (ICM) has proven more effective in detecting AF than standard clinical follow-up, at a rate as high as 30% after 3 years of continuous monitoring [3]. AAA is another possible cause of ischemic strokes, with higher risk in the presence of a mobile or intraluminal protruding thrombus or ulceration [4]. Accordingly, classification systems of stroke etiology, such as the “ASCOD Phenotyping of Ischemic Stroke”, generally consider an aortic plaque definitely pathogenic only when these specific conditions are met [5]. However, recent data have shown that also aortic plaques without high-risk features are associated with ischemic stroke and should be considered in the evaluation of cryptogenic stroke [6]. Atherosclerotic disease of the carotid arteries causing a stenosis inferior to 50% may be an additional explanation of the cause of stroke in cryptogenic stroke [7]. In support of the causal link between cryptogenic stroke and NCP, recent studies have shown that the prevalence of plaques ipsilateral to a cerebral infarction is higher than on the contralateral side [8, 9].

In a cohort of cryptogenic stroke patients who received an ICM, the aims of this study were: (1) using data from ICM and head–neck CT angiography (CTA) re-evaluation, to assess the prevalence of subclinical AF, AAA, and NCP and to reclassify stroke etiology accordingly; (2) to compare the clinical features of patients with or without a reclassified stroke etiology.

## Methods

The study was carried out at the stroke unit of a primary stroke center (Luigi Sacco University Hospital, Milan, Italy), admitting patients from several municipalities in the metropolitan area of Milan. Patients with a diagnosis of ischemic stroke admitted to the stroke unit between January 26, 2018, and February 26, 2021 were included in the study. During hospitalization, patients received standard assessments and care according to current European and Italian guidelines [10, 11]. After an extensive evaluation including blood tests, brain imaging, head and neck CTA, in-hospital ECG monitoring (at least 24 h), and echocardiography, a diagnosis of cryptogenic stroke was made according to the following criteria: brain infarct demonstrated by CT or MRI that is not lacunar in appearance; no extracranial or intracranial atherosclerosis causing ≥ 50% luminal stenosis in arteries supplying the ischemic area; no aortic arch atherosclerotic plaque with mobile or protruding thrombus; no major-risk cardioembolic source; no other specific causes of stroke (e.g., arteritis, dissection, migraine/vasospasm, drug misuse) [12].

At the time of the index stroke, patients’ demographic and clinical features (age, gender, body mass index, hypertension, diabetes mellitus, dyslipidemia, coronary artery disease, previous stroke or transient ischemic attack, smoking habit) were recorded. We assessed pre-stroke disability using the modified Rankin scale (mRS) [13], and stroke severity using the National Institutes of Health Stroke Scale (NIHSS) [14]. Brain imaging characteristics were also evaluated, including infarct size, involvement of multiple vascular territories, and involvement of posterior circulation. An experienced cardiologist measured echocardiographic parameters (left atrial diameter and volume, left atrial diameter index, left ventricular ejection fraction, and presence and degree of mitral insufficiency) [15]. A left atrial diameter index ≥ 3 cm/m^2^ was considered indicative of the presence of atrial cardiopathy [16].

Patients with a cryptogenic stroke were proposed to receive ICM if no contraindication to anticoagulant therapy was present at the time of implant, and if the patient was able to pursue the required follow-up (e.g., attend regular cardiologic and neurologic out-patient services; correctly manage the home device). Follow-up was carried on until May 26, 2021. During this time, all transmissions with AF events notified by the device (≥ 2 min in duration), as per device related algorithms, were reviewed by an experienced cardiologist to rule out false alarms and eventually make the diagnosis of AF. Diagnostic criteria for ICM-detected subclinical AF, the devices used, and the implantation procedures have been thoroughly described in previous works [17].

Baseline CTA was re-evaluated by an experienced radiologist, who assessed the presence and thickness of aortic arch plaques and the presence and degree of stenosis of carotid plaques according to NASCET (North American Symptomatic Carotid Endarterectomy Trial) criteria [18, 19]. The radiologist was blinded to patients’ clinical details and brain parenchymal imaging (thus, he was unaware of side, morphology, and localization of the ischemic lesion). For strokes with a unilateral brain infarction involving the carotid circulation, carotid atherosclerosis was considered possibly pathogenic in the presence of a plaque causing < 50% stenosis. AAA was considered possibly pathogenic if the plaque thickness was ≥ 4 mm [5]. Since aortic plaques with ulceration or intraluminal thrombus were already considered pathogenetic during the initial workup [4], only patients with milder AAA were included. Cryptogenic stroke etiology was eventually reclassified as

- “cardioembolic”, if AF was detected, but neither AAA nor an ipsilateral NCP were present;
- “atherosclerosis” if no AF was detected, and AAA and/or ipsilateral NCP were present;
- “mixed” if both AF and at least one between AAA and ipsilateral NCP were present;
- “true cryptogenic” if no AF, AAA, or ipsilateral NCP were detected.

### Statistical analyses

We used the Shapiro–Wilk *W* test to assess the normality of distribution for all quantitative measures. Descriptive statistics were reported as medians and interquartile range (IQR) for not normally distributed quantitative variables, or frequencies (%) for categorical variables. To determine whether patients who received an ICM were representative of the whole cryptogenic stroke cohort, we compared clinical characteristics of included and excluded patients using the Chi-square test (for categorical variables) and the Mann–Whitney *U* test (for quantitative variables). We compared clinical and echocardiographic characteristics of patients with reclassified stroke etiology and those with true cryptogenic stroke after ICM and CTA re-assessment using the Chi-square test (for categorical variables) and the Mann–Whitney *U* test (for quantitative variables). Since we only found one variable being significantly different between the two groups (i.e., age), we decided not to perform multivariable analyses. We used a *p* value threshold of 0.05 to ascertain statistical significance for all analyses. We performed all statistical analyses using the SPSS software (version 22; IBM Corp.).

## Results

**Fig. 1:**
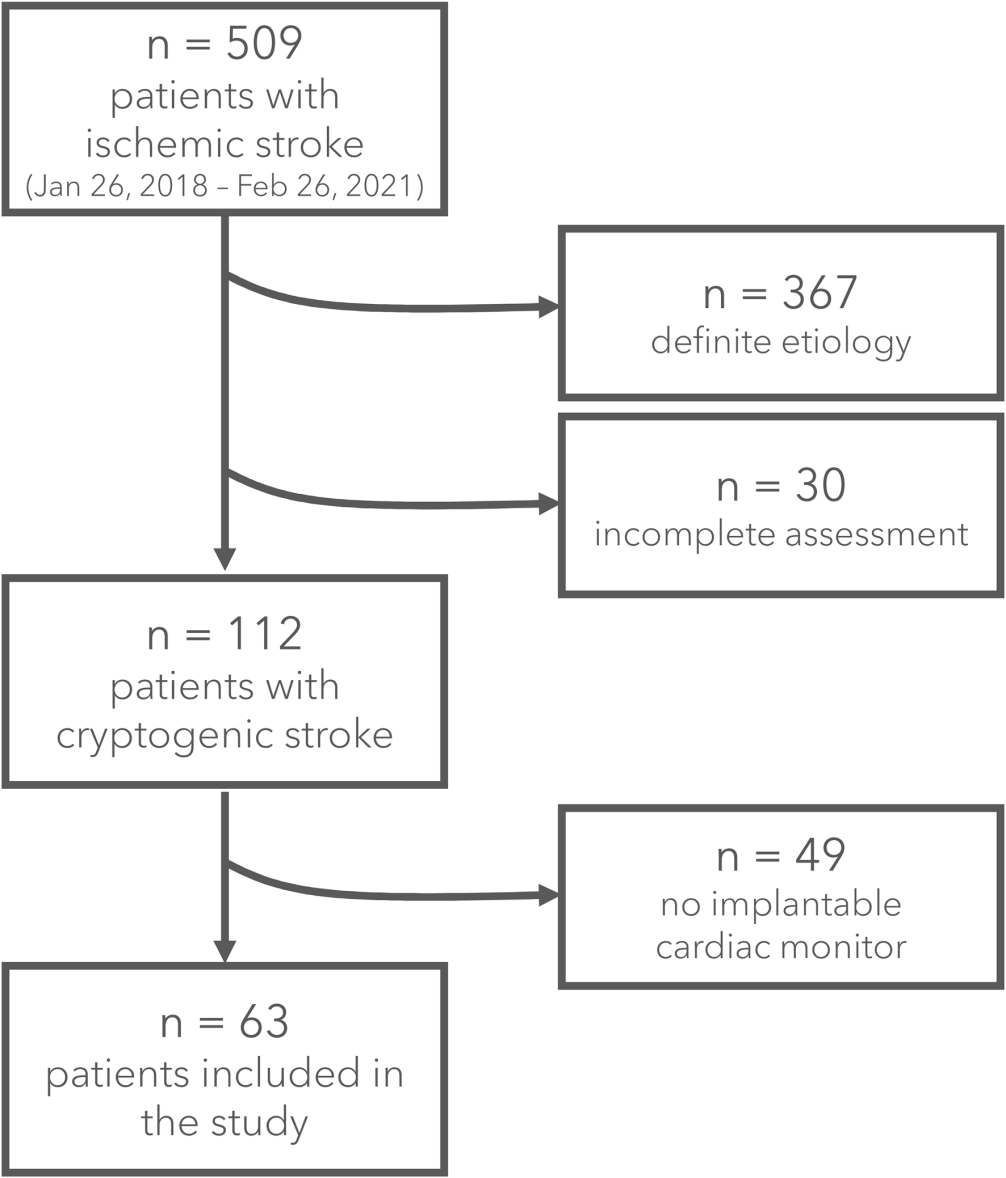
Study flowchart

Out of 509 patients with a definite diagnosis of ischemic stroke admitted to our center during the study period, 30 (6%) were excluded for an incomplete diagnostic assessment, 367 (72%) had a definite cause recognized after clinical workup, and 112 (22%) were defined cryptogenic. Sixty-three of them (56%) received an ICM and were included in the study (as shown in Fig. 1).

Median age of patients included in the study was 71 years (IQR 64–78), 43 (68%) were male, 6 (10%) had pre-stroke disability, and median NIHSS score at stroke onset was 2 (IQR 1–5). Compared to the patients included in the study, those who did not receive an ICM (*n* = 49, 44%) were overall older, had higher rates of diabetes mellitus, higher rates of pre-stroke disability, and higher median NIHSS scores at baseline (as shown in Supplementary Table 1).

### Subclinical atrial fibrillation detection and CTA re-assessment

**Fig. 2:**
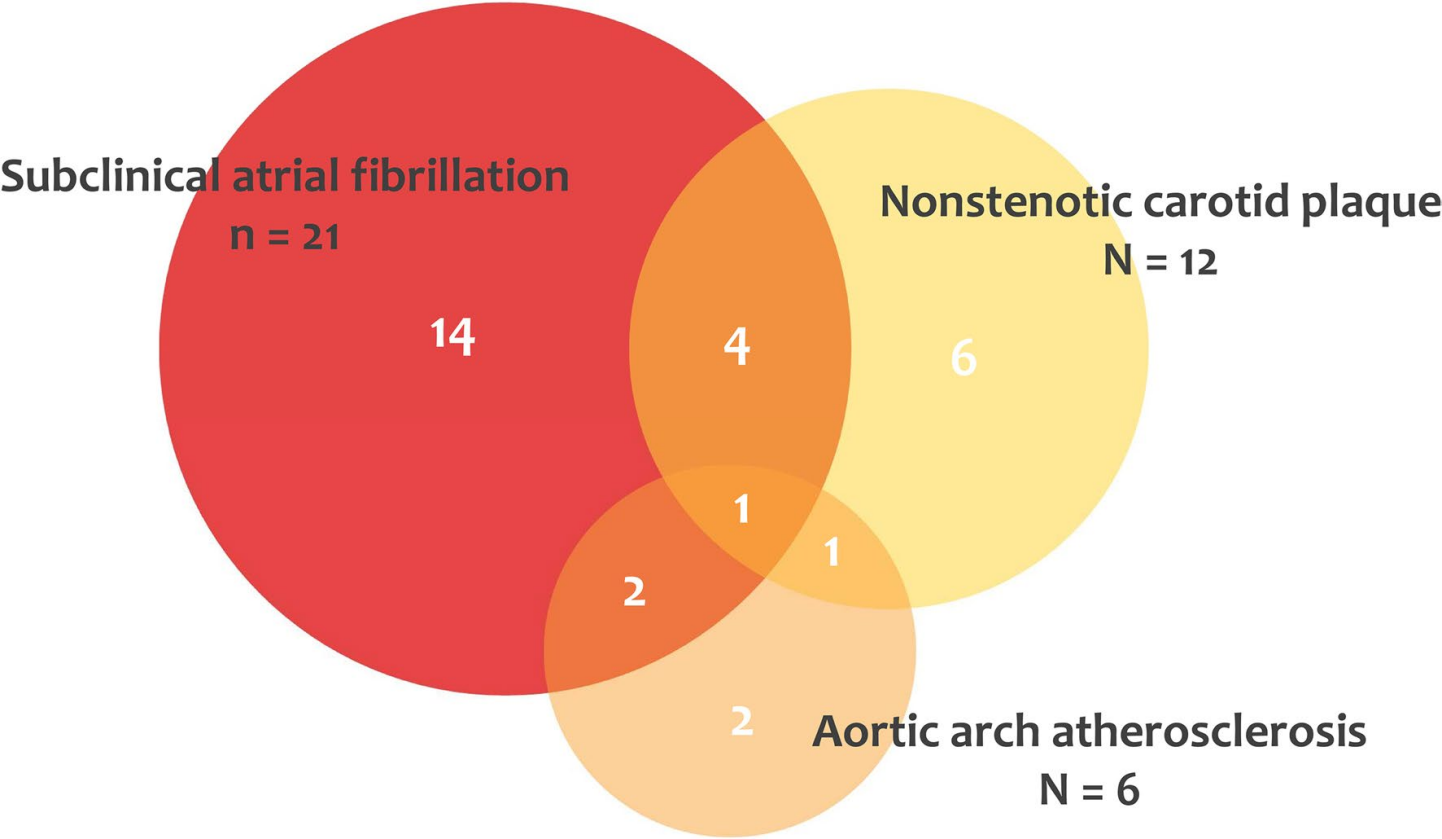
Subclinical atrial fibrillation detection and computed tomography angiography re-assessment. Covert atrial fibrillation was diagnosed on implantable cardiac monitoring over a median follow-up time of 15 months. Aortic arch atherosclerosis was considered relevant in case of a plaque thicker than 4 mm; nonstenotic carotid plaque was considered relevant when ipsilateral to a stroke in internal carotid artery territory

The median length of cardiac monitoring follow-up after stroke was 15 months (IQR 9–24). During this time, 21 patients out of 63 (33%) were diagnosed with AF, with a median time from implantation to detection of 5 months (IQR 2–9). After aortic arch and neck CTA re-assessment, we found 12 patients (19%) with a NCP ipsilateral to the index stroke, and 6 patients (10%) with aortic plaques thicker than 4 mm. In 8 patients we found more than one possible stroke etiology (as shown in Fig. 2). The percentage of patients who had at least one between NCP or AAA was 33% (7 out of 21) in the group of patients with an ICM-based diagnosis of AF, and 21% (9 out of 42) in those without (*p* = 0.306).

### Reclassified stroke etiology

**Fig. 3:**
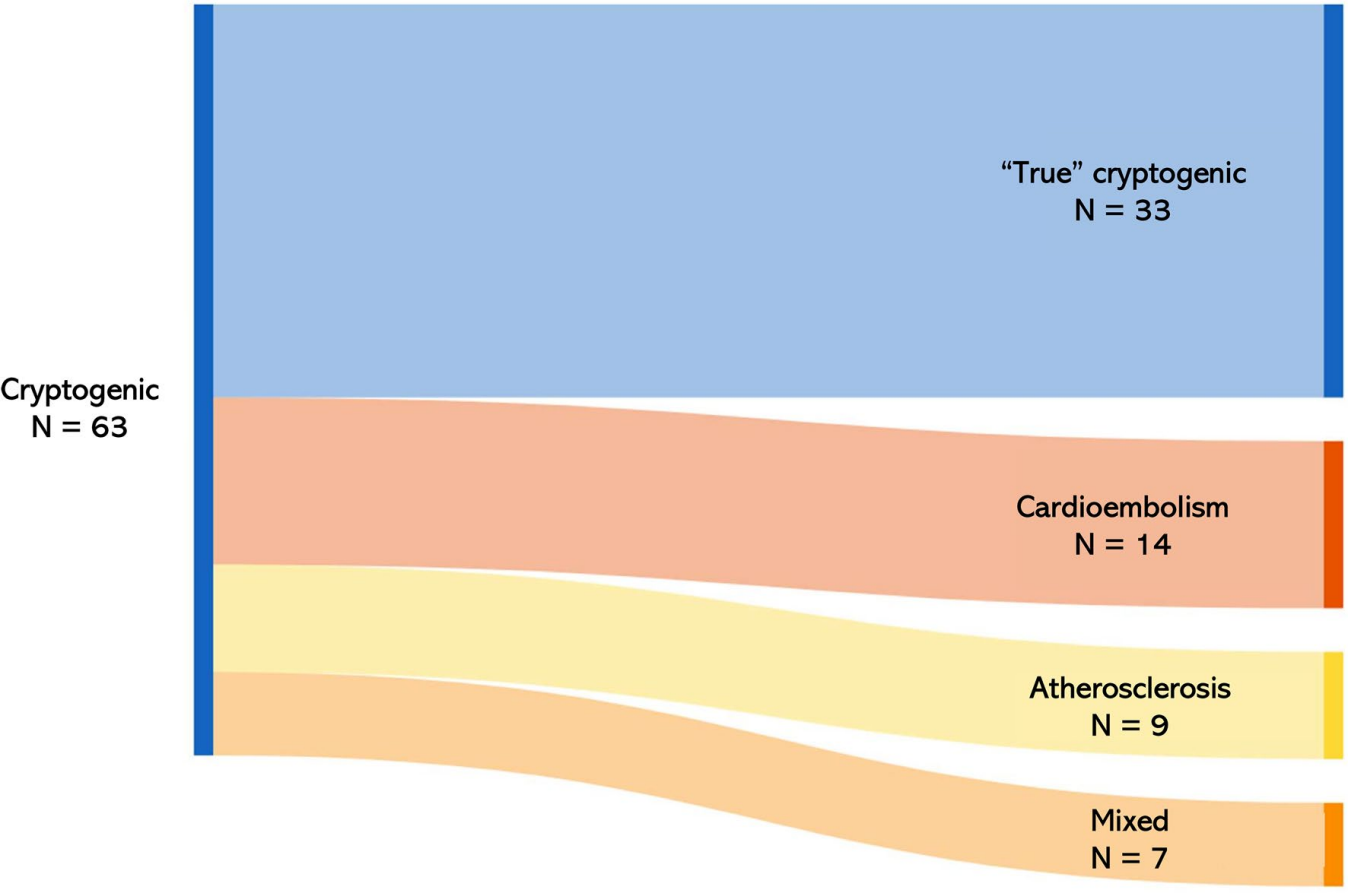
Sankey diagram of cryptogenic stroke reclassification. Made with SankeyMATIC (sankeymatic.com/build/)

Based on the pre-specified criteria, we reclassified stroke etiology in 30 patients (48%): 14 (22%) had cardioembolic, 9 (14%) had atherosclerotic, and 7 (11%) had mixed (cardioembolic + atherosclerotic) stroke etiology after prolonged cardiac monitoring and CTA re-assessment. Thirty-three patients (52%) had still no identifiable stroke etiology and were thus classified as “true cryptogenic” (as shown in Fig. 3).

Compared to patients with reclassified stroke etiology, “true” cryptogenic stroke patients were younger (median age 67 vs. 76 years; *p* = 0.001), but we did not find statistically significant differences in vascular risk factors, baseline stroke characteristics, and echocardiographic features (Table 1); 1 patient in each subgroup had left atrial diameter index ≥ 3 cm/m^2^.

**Table 1:** Characteristics of patients with reclassified stroke etiology vs. true cryptogenic stroke

	All patients	Reclassified stroke etiology	“True” cryptogenic	*p*-value
	*n* = 63	*n* = 30	*n* = 33	
Age, years	71 (64–78)	76 (69.5–82)	67 (57.5–74.5)	0.001
Gender (men)	43 (68.3)	21 (70.0)	22 (66.7)	0.777
Body mass index, kg/m^2^	26.5 (23.4–29.4)	27.2 (23.1–29.1)	25.8 (23.7–30.0)	0.901
Hypertension	47 (74.6)	25 (83.3)	22 (66.7)	0.129
Diabetes mellitus	14 (22.2)	4 (13.3)	10 (30.3)	0.106
Dyslipidemia	44 (69.8)	22 (73.3)	22 (66.7)	0.565
Smoking	16 (25.4)	8 (26.7)	8 (24.2)	0.867
History of coronary artery disease	14 (22.2)	9 (30.0)	5 (15.2)	0.157
Previous stroke or TIA	13 (20.6)	8 (26.7)	5 (15.2)	0.259
mRS before stroke > 1	6 (9.5)	4 (13.3)	2 (6.1)	0.326
Stroke characteristics				
NIHSS score at baseline	2 (1–5)	3 (1–6)	2 (1–4)	0.297
Multiple territory stroke	8 (12.7)	2 (6.7)	6 (18.2)	0.170
Posterior circulation stroke	18 (28.6)	8 (26.7)	10 (30.3)	0.750
Lesion size (maximal diameter), mm	23 (13–37)	24 (12–38)	19 (14–35)	0.751
Echocardiography				
Atrial diameter, mm	39 (36–42)	40 (37–43)	38 (35–42)	0.268
Atrial volume, cm^3^	53 (43–64)	54 (48–66)	50 (45–60)	0.170
Left atrial diameter index	2.2 (1.8–2.4)	2.2 (1.8–2.5)	2.2 (1.9–2.3)	0.751
Left atrial diameter index ≥ 3 cm/m^2^	2 (3.2)	1 (3.3)	1 (3.0)	NA
Left ventricular ejection fraction	62 (58–65)	62 (60–65)	61 (57–65)	0.529
Mitral valve insufficiency	35 (58.3)	18 (64.3)	17 (53.1)	0.382

Values are expressed as median (interquartile range) for continuous variables, and as number of cases (percentage) for categorical variables. Statistical test used: Mann–Whitney *U* test for continuous variables and Chi-square test for categorical variables

*TIA* transient ischemic attack, *mRS* modified Rankin scale, *NIHSS* National Institutes of Health Stroke Scale

## Discussion

Among patients diagnosed with cryptogenic stroke who underwent etiologic reclassification using data from ICM and CTA, one-third were diagnosed with AF over a median ICM follow-up time of 15 months, one in five had NCP of the internal carotid artery ipsilateral to the index stroke, and one in ten had aortic arch atherosclerotic disease. Based on the pre-specified criteria, stroke etiology was reclassified in nearly half of patients that were included in our study. Indeed, we found that cardioembolism was the most common possible etiology (22%), followed by atherosclerosis (14%) and mixed (cardioembolism plus atherosclerosis) cause (11%). Patients with true cryptogenic stroke had younger age compared to those with reclassified etiology, with no further substantial difference in cardiovascular risk factors.

Our study confirms the frequent occurrence of unrecognized stroke etiology among patients hospitalized for acute ischemic stroke, with up to one-fourth having no definite recognizable cause at discharge even after an extensive diagnostic workup. In line with other results from previous observational studies, prolonged cardiac monitoring by means of ICM allowed the detection of subclinical AF in one-third of our cryptogenic stroke patients, with roughly half of the diagnoses made in the first 5 months after the implantation [17]. Indeed, the ICMs have specific AF detection algorithms allowing better estimation of the arrhythmic burden as compared with intermittent monitoring approaches [20]. Prolonged cardiac monitoring is now recommended to improve the detection rates of subclinical AF after a stroke of undetermined origin, and to increase the percentage of patients who could benefit from secondary prevention with anticoagulants [21]. Nevertheless, it is difficult to establish a causal association between the index stroke and subclinical AF detected long after, since non-negligible rates of subclinical AF have been observed in high-risk stroke-free patients [22]. However, some studies suggest that subclinical AF should be seen more as a biomarker of a cardioembolic event and atrial cardiopathy than an established stroke source [23]; moreover, ICM seems effective to increase anticoagulant initiation and to lower stroke recurrence rates in patients with cryptogenic stroke [24]. We also found high rates of potentially pathogenetic atherosclerotic stigmata, namely NCP or AAA, in our cryptogenic stroke patients, not infrequently coexisting with subclinical AF detected through ICM: this finding is not surprising, since AF and atherosclerosis share common risk factors [25]. In the last few years, several randomized controlled trials have failed to demonstrate a benefit of direct oral anticoagulants for the secondary prevention of embolic stroke of undetermined source [26, 27]. Despite a causal association between NCP and stroke might be difficult to establish, the high rates of NCP and AAA detected in our cryptogenic stroke patients, together with evidence from previous studies, underline the need to also consider the presence of potential atherosclerotic sources in the management of stroke of undetermined origin [28, 29]. Moreover, the most appropriate choice for the secondary prevention of stroke when multiple potential sources are recognized is yet to be established, as more studies are needed to optimize a therapeutic strategy tailored on a patient’s own medical history and risk profile.

Overall, we were able to identify a possible source of stroke in nearly half of the patients included in our study. This result underlines a key concept in stroke pathophysiology, meaning that the definition of cryptogenic stroke is intrinsically related to the effort put in the diagnostic workup [30]. On the other side, half of patients had neither cardioembolic nor atherosclerotic potential stroke sources recognized, despite long-term cardiac monitoring and CTA re-assessment. These patients were younger compared to patients with a reclassified stroke but shared similar cardiovascular risk profiles. Probably, other sources of cardioembolism might be recognized as a possible cause in a percentage of these patients, although our echocardiographic data (left atrial structural parameters being within normal limits in most of our patients) suggest that the so-called atrial cardiopathy does not play a major role in these patients’ stroke pathophysiology. Some studies have hypothesized mechanisms other than atherosclerosis and cardioembolism, such as hypercoagulable states (e.g., in case of covert malignancies), artery dissection, and vasculitis that might be rather prevalent in young stroke patients [31], which is not however the case of our sample. We think that longitudinal studies are needed to evaluate the risk profile, in terms of stroke relapse, cardiovascular events, and mortality, of patients with true cryptogenic stroke compared to patients having a possible stroke etiology.

The major strength of our study is its novelty and the extensive diagnostic workup carried out, since we were able to obtain CTA imaging of both internal carotid arteries and aortic arch, as well as collecting a complete long-term cardiac rhythm monitoring using an ICM strategy in all patients. Our study has also several important limitations. First, this is a retrospective analysis from a single-center cohort, thus hampering the generalizability of our results. Moreover, the number of patients included (considerably affected by the COVID-19 pandemics outbreak) [32, 33] did not allow us to draw definitive conclusions, as our results need to be replicated in larger cohorts. For the same reason, we decided not to perform longitudinal risk analyses (despite we think they would be of great interest), and we did not compare baseline characteristics of patients between different subgroups (i.e., subclinical AF *vs.* atherosclerotic *vs.* true cryptogenic). Nevertheless, the present study should be considered as hypothesis-generating, since our results suggest that a detailed diagnostic profiling might shed new insight in the pathophysiology of cryptogenic stroke. We recognize that the exclusion of a relevant number of cryptogenic stroke patients who were not fit enough to undergo long-term cardiac monitoring (they were older and had higher rates of pre-stroke disability compared to the patients included) must be accounted as a potential weakness in the interpretation of our results, as our cohort is probably not representative of the entire cryptogenic stroke population. The cohort is however likely representative of the patients who can be implanted in clinical practice. Moreover, due to the retrospective nature of the present study, we were not able to assess the prevalence of patent foramen ovale in true cryptogenic stroke and reclassified stroke etiology patients, even though we acknowledge it would have been of interest. Finally, we acknowledge that the exclusion of nonstenotic vertebral artery plaques from the reclassification criteria might constitute a possible bias in the interpretation of results; nevertheless, to our knowledge, no study has previously assessed the pathogenic potential of nonstenotic plaques in posterior circulation stroke.

In conclusion, our results suggest that a possible covert stroke source might be recognized in a half of patients with a diagnosis of cryptogenic stroke through long-term cardiac monitoring and careful CTA re-assessment. The relatively frequent finding of multiple possible stroke etiologies emphasizes the need for a careful and patient-tailored diagnostic and therapeutic pathway. Accordingly, more studies are needed to ascertain the pathogenic potential of NCP, AAA, and covert AF diagnosed through ICM, and to achieve a deeper understanding of the genetic and acquired pathophysiologic background of those younger patients with a “true” cryptogenic stroke.

## Supplementary Information

Below is the link to the electronic supplementary material.

Supplementary file1 (PDF 75 KB)

## Data Availability

The data that support the findings of this study are available from LP upon reasonable request.
