# Supplementary material for: Etiologic reclassification of cryptogenic stroke after implantable cardiac monitoring and computed tomography angiography re-assessment
Source: J Neurol. 2022 Sep 13;270(1):377–85. doi: 10.1007/s00415-022-11370-x (PMC9469058; doi:10.1007/s00415-022-11370-x)
Supplement: Supplementary file 1 — Supplementary file1 (PDF 75 KB) [file 415_2022_11370_MOESM1_ESM.pdf]

Research Article
Journal of Neurology

Etiologic reclassification of cryptogenic stroke after implantable cardiac monitoring and computed tomography angiography reassessment

Francesco Mele,[1,*] Giuseppe Scopelliti,[2,3,*] Arianna Manini,[2,4] Carola Ferrari Aggradi,[2] Matteo Baiardo,[2] Marco Schiavone,[5] Maurizio Viecca,[5] Andrea Ianniello,[6] Pierluigi Bertora,[1,2] Giovanni Forleo,[5] Leonardo Pantoni [1,2]

1. Neurology Unit, Luigi Sacco University Hospital, Milan, Italy.
2. Department of Biomedical and Clinical Sciences, University of Milan, Milan, Italy
3. Univ. Lille, Inserm, CHU Lille, U1172 - LilNCog - Lille Neuroscience & Cognition, Lille, France
4. Department of Pathophysiology and Transplantation, “Dino Ferrari” Center, University of Milan, Milan, Italy.
5. Cardiology Unit, Luigi Sacco University Hospital, Milan, Italy
6. Radiology Unit, Luigi Sacco University Hospital, Milan, Italy

* The first two authors equally contributed to the paper

Corresponding Author:

Leonardo Pantoni, MD, PhD

Department of Biomedical and Clinical Sciences, University of Milan

Via Giovanni Battista Grassi, 74, 20157, Milano, Italy

Phone: +39-02-50319865; e-mail: leonardo.pantoni@unimi.it

	ICM patients n = 63	No ICM patients n = 49	p-value
Age, years	71 (64-78)	78 (68-84)	**0.019**
Gender (men)	43 (68.3)	30 (61.2)	0.439
Body mass index	26.5 (23.4-29.4)	26.1 (24.7-28.5)	0.955
Hypertension	47 (74.6)	36 (73.5)	0.892
Diabetes mellitus	14 (22.2)	20 (40.8)	**0.034**
Dyslipidemia	44 (69.8)	30 (61.2)	0.339
mRS before stroke > 1	6 (9.5)	12 (24.5)	**0.032**
NIHSS score at baseline	2 (1-5)	5 (3-12.5)	**<0.001**

Supplementary table 1. Characteristics of cryptogenic stroke patients with ICM (included in the study) and without ICM (excluded from the study). Values are expressed as median (interquartile range) for continuous variables, and as number of cases (percentage) for categorical variables. Statistical test used: Mann-Whitney U test for continuous variables and chi-square test for categorical variables. ICM = implantable cardiac monitor; mRS = modified Rankin scale; NIHSS = National Institutes of Health Stroke Scale
